# The Incidence of Dementia in England and Wales: Findings from the Five Identical Sites of the MRC CFA Study

**DOI:** 10.1371/journal.pmed.0020193

**Published:** 2005-08-23

**Authors:** Fiona Matthews, Carol Brayne, Medical Research Council Cognitive Function and Ageing Study Investigators

**Affiliations:** **1**MRC Biostatistics Unit, Institute of Public Health, University Forvie Site, Robinson Way, Cambridge, United Kingdom,; **2**Department Public Health and Primary Care, Institute of Public Health, University Forvie Site, Robinson Way, Cambridge, United Kingdom; University of MelbourneAustralia

## Abstract

**Background:**

Although incidence of dementia is known to vary between nations, variation within country has not been explored because most incidence studies are single site or have insufficient numbers to compare sites. Few countries have conducted multisite incidence studies in order to facilitate national comparisons. This study aims to provide robust measures of the variation of the incidence of dementia across sites within England and Wales and produce overall estimates by age and sex.

**Methods and Findings:**

The Medical Research Council Cognitive Function and Ageing Study used identical methodology in five diverse sites across the United Kingdom, each with different risk patterns and mortality rates. Incidence has been estimated using likelihood-based methods between the first two waves of interviews. Incidence rates rise with age, particularly above the age of 75 y, from 7.4 (95% confidence interval, 3.6–16.1) per 1,000 person years at age 65–69 y to 84.9 (95% confidence interval, 63.0–107.8) per 1,000 person years at age 85 y and above. The rate of increase for both sexes is marked, and continues into the oldest age groups. Hence, it is estimated that approximately 180,000 new cases of dementia occur in England and Wales each year. There is no convincing evidence of variation across sites, and incidence rates do not reflect the variations in the prevalence of possible risk factors in these sites.

**Conclusion:**

There is no evidence, within England and Wales, of variation in dementia incidence across sites. Dementia incidence rates do not tail off at the oldest ages.

## Abstract 2

No evidence for variation in dementia incidence between areas with different vascular disease risk or between men and women, nor for reduced indidence amongst oldest age groups.

## Introduction

Dementia remains an incurable condition of major significance to the world's ageing populations. There have been numerous studies of the prevalence, incidence and natural history of dementia though there have been very few which compare diverse locations. Most studies are based on a single site (e.g., Rotterdam, The Netherlands [1], Framingham, Massachusetts, United States [2], East Boston, Massachusetts, United States [3], and Cache County, Utah, United States [4]) or take small population samples from multiple sites (the Canadian Study of Health and Aging [5] and the Italian Longitudinal Study on Aging [6]). Incidence studies of dementia are particularly valuable for less-biased comparison of disease occurrence, pointing to aetiological investigation, as well as being essential for policymakers. Comparisons of incidence estimates are hampered by any variation in methodology. Despite this, researchers frequently attempt to assess whether rates in a given study are similar to those provided elsewhere [7]. Although such estimates, on the whole, seem reasonably consistent across the more developed regions of the world, there have been widely divergent estimates reported within the United States (East Boston study [3] versus Chicago, Illinois [8], in which the disorder definition is different, but even between Framingham [2] and the Northern Manhattan [New York] Aging Project [9] in which the disease criteria are identical.) These variations have been dismissed as being due to the variation in methodology, which may be true, but without identical methodology, this cannot be known.

There are substantial differences in possible risk factors for dementia within the developed world. Such chronic disease risk factor variation is thought to be responsible for the wide variation seen in other diseases of older age such as cancer and cardiovascular disease (e.g., differences between North and South Europe). The only systematic examinations across sites with identical methodology and sufficient numbers for comparison are between two less-developed regions and the United States: the Ibadan, Nigeria–Indianapolis, Indiana, United States study [10] and the rural India–United States study [11]. These did show substantial differences across sites, which indicate that variation, if large, can be detected. No such similar exercise has been conducted within countries with diverse risk patterns. Given the available evidence on vascular risk factors for dementia, strengthened by the published single site longitudinal studies, and the marked variation in vascular risk across Europe and within European sites [12], there could be parallel variation in the incidence of dementia. The whole thrust of the prevention of vascular risk for dementia (e.g., Forette et al. [13]) is based on the premise that dementia incidence could be reduced if vascular risk were better controlled. It is reasonable therefore to compare sites with known variable vascular risk to assess whether there is variation in the incidence of dementia. Previously we have shown that areas across England and Wales vary considerably with regards to their vascular risk patterns [14]. Cardiovascular risks varied by centre from high values in Newcastle, England, and Gwynedd, Wales (angina 16%, intermittent claudication 4%, heart attacks 12%, and stroke 8%) to low prevalence in Cambridgeshire and Oxford, England (angina 11%, intermittent claudication 2%, heart attacks 9%, and stroke 6%). Nottingham, England, generally lies in between, however it shows a high stroke rate (8%) and low intermittent claudication rate (0.5%).

Whether or not there is variation in the incidence of dementia across sites, it is of particular importance for policy to understand the occurrence and natural history of dementia in the over 80-y-olds. This is the population group expanding at the greatest rate at present. In the United Kingdom over the past decades, a number of small field-based studies have reported incidence in the very old (e.g., the Cambridge City over 75 cohort [15,16], Melton Mowbray [17], and Gospel Oak [18,19]). The limited number of respondents in each of these single centre studies in the oldest age groups has hampered the examination of incidence in these groups. Those estimates available in the published literature are based on combined analyses of diverse studies [20].

The Medical Research Council Cognitive Function and Ageing Study (MRC CFAS) is a multi-site study in the United Kingdom and has already published findings on the prevalence of dementia across six sites [21]. No systematic differences were found across the five identical sites within the study. It is known that there is considerable variation in mortality across the country and in the sites that are represented in this study [22–24]. Thus the absence of variation in prevalence could be masking an increased incidence allied to an increased mortality in the northern sites. This can only be investigated through longitudinal studies. Here we present data from the 2-y follow-up of the baseline population, to compare incidence estimates of dementia across five methodologically identical sites with widely differing risk profiles.

One of the sites, the Liverpool centre, was funded earlier and had a slightly different design; the Liverpool incidence study is described in detail elsewhere and not included further here [25].

## Methods

MRC CFAS is a population-based study of individuals aged 65 y and over living in the community, including in institutions; it has a two-phase two-wave design, with the waves 2 y apart. Ethical approval for the study was obtained in each site. The prevalence results have already been discussed in detail [21]. Two sites were rural—Cambridgeshire and North Wales (Gwynedd)—and three urban—Nottingham, Newcastle, and Oxford. These were selected both for known possible chronic disease risk factors–the north has high vascular risk load—and for epidemiological expertise in conducting population-based research.

### Baseline Sample

The population sample was drawn from the Family Health Service Authorities lists covering specific geographically defined areas. These are population-based registers of the general practitioners, which provide nearly complete population enumeration including in institutions. Random selection was carried out to recruit at least 1,250 individuals in each centre in each of the age groups 65 to 74 y and 75 y and above. Population sampling levels varied from 1 in 2.4 individuals in Cambridgeshire in the older age group, to 1 in 14 in Newcastle.

### The Approach and Sequence of Interviews Relevant to Calculation of Incidence

Individuals selected for the study were approached either through an introductory letter from their general practitioners or from the local principal investigator with a follow-up visit or telephone call to seek permission to visit and explain the study. Those who agreed to participate were asked for informed consent before proceeding to interview. When an individual was unable to give informed consent, a proxy (relative or caregiver) was approached. The first interview was a screening interview, which was followed by a more intensive assessment of a subsample of the study population. After 2 y a re-screen of individuals who were not selected for assessment at baseline was undertaken, followed by a selection of a further assessment sample. Those who had been previously assessed were interviewed with a combined screen and assessment interview. The study design to the end of wave 2 is shown in Figure 1. Further interviews with informants, interim interviews, and interviews after 2 y have been undertaken but are not used in the analysis presented here.

**Figure 1:**
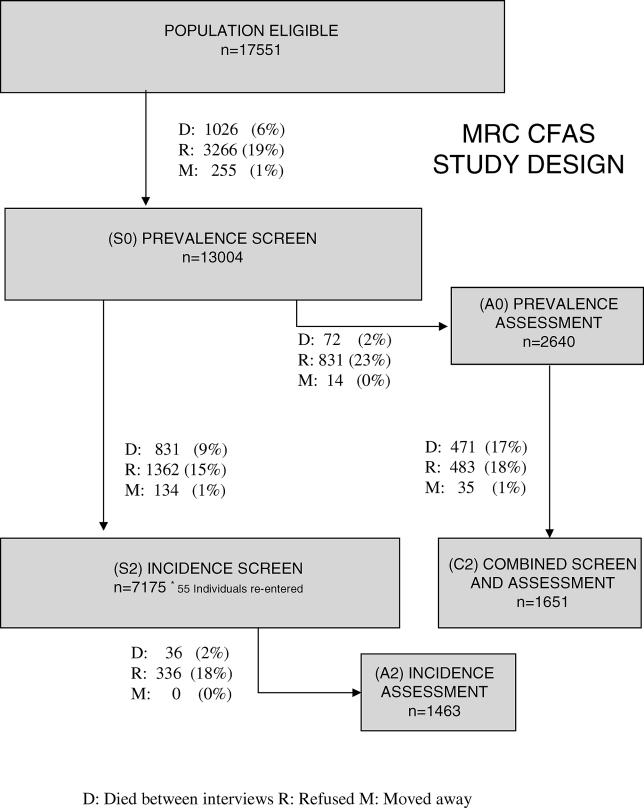
Flow Chart of Individuals Contributing to the Incidence Analysis D indicates died between waves, R indicates refused, and M indicates moved away from the study areas between waves.

### The Interviewers

#### Baseline and incidence screen

Interviewers were recruited locally for each site, with a range of ages and backgrounds, mainly professions allied to medicine. They were all trained in interviewing and the computer skills necessary for administration of the computerised interview. The local interviewers were trained by local coordinators who had been trained by the national coordinator to ensure consistency. Repeat interviewing was not carried out. Monitoring of the quality of interviews with feedback was undertaken throughout the study. Intermittent audio taping of interviews, with all local interviewers meeting regularly to listen and rate each others' tapes, was carried out to ensure continuing comparability across sites. The national coordinator visited all sites regularly.

#### Baseline and incidence assessment/combined screen assessment

Interviewers were recruited locally and nationally for each site, and included psychologists, psychiatrists, registered nurses, and others with similar backgrounds, all trained by the National Coordinator. Interviewers regularly audiotaped interviews for quality checks and took part in between-centre meetings with joint rating and discussion of discrepancies during the study period to ensure comparability across sites.

### The Interviews

#### Prevalence screen [19.12.1990 to 13.6.1994]

The prevalence screen interview (S0) lasted an hour and contained basic information on residence, marital status, social class, and main occupation during working life [26]; social and service contacts [27]; physical health and well-being, including vascular risk factors [28,29]; activities of daily living as measured by the Townsend scale [30,31]; organicity items from the Geriatric Mental State, a standardised psychiatric interview designed to detect dementia, depression, and other mental illness in the older population, to which an algorithmic programme (AGECAT [Automated Geriatric Examination Computer Assisted Taxonomy]) is applied to create a diagnosis, including items for the organicity scale (O0 to O5, with O3 and above indicating dementia) [32]; Mini-Mental State Examination (MMSE) with additional items [33,34]; and regular medication (prescribed and over the counter).

#### Prevalence Assessment [26.2.1991 to 21.9.1994]

Undertaken in approximately 20% of those in S0, the prevalence assessment interview (A0) has a clinically based content and is built on the Geriatric Mental State (GMS) version B3, which focuses on dementia and depression [32]. It allows for the generation of the dementia measure of the study—the case-level organicity status (O3 and above, with incorporation of the other diagnostic algorithms to take into account comorbidity such as depression and anxiety).

#### Incidence screen [22.2.1993 to 16.7.1996]

After 2 y, respondents who were not previously assessed were approached for re-interview. The incidence screen interview (S2) was substantially the same as S0, with the omission of stable items, such as earlier occupation, and with modification of physical health measures to collect information on new events since the last interview.

#### Incidence assessment [28.6.1993 to 29.7.1996]

The incidence assessment interview (A2) followed on in a 20% selected sample from S2 and was the same as A0.

#### Combined screen assessment [13.4.1993 to 25.7.1996]

All individuals who had previously been assessed were approached for a combined screen and assessment (C2). This was a merged interview with collection of key variables from the screen, change in physical health, and activities of daily living, along with the diagnostic component of the assessment interview.

#### Priority mode

Interviewers at the S0 and S2 stages were instructed to complete interviews whenever possible, but when individuals were unable to answer the first few questions accurately (due to disorientation in time and space), there was a systematic skip such that the interview was truncated to a small set of priority questions that included the organicity items of the GMS [32] and the MMSE [33]. Interviewers were also able to select the priority mode if they felt the interview was jeopardised and a complete interview would be impossible.

### Selection for Assessment

Four of the sites used identical sampling fractions from the screening interview data for assessment at prevalence (shown below). In Cambridge, the density was reduced after approximately one year due to higher numbers of the sample population eligible for assessment. The sampling fractions varied by age at screen and cognitive ability at that interview. These sampling fractions were programmed into the interview. The sampling groups were age group (65–74 y or 75 y and above), ability group (AGECAT O3+; AGECAT O0–O2 and MMSE less than 21 or missing; AGECAT O0–O2 and MMSE 21–25; and AGECAT O0–O2 and MMSE 26–30). The sampling groups for selection to the incidence assessment were similar, modified slightly based on experience, with different sampling fractions and with the ability group calculated at the incidence screen. There was a change in the sampling fractions during the second-wave selection process because too many were being selected for assessment; this change was programmed into the software with a version label. The whole scoring spectrum was eligible for selection to assessment at both waves with theoretical sampling fractions varying from 1:1 to 1:15 at both waves. The exact weights can be found on the MRC CFAS website (http://www-cfas.medschl.cam.ac.uk/data_and_analysis.htm#Weights). For analysis, empirical weights have been used as these have been shown to be more robust [35].

### Flagging

The whole sample was flagged at the National Health Service Central Register, which collects all death registrations from England and Wales. Quarterly updates of the vital status of participants were therefore possible, not just those seen at follow-up.

### Study Diagnosis

Diagnosis was based on the GMS B3 organicity case level generated by AGECAT as described above, equivalent to *Diagnostic and Statistical Manual of Mental Disorders, Third Edition, Revised* diagnoses [32,36]. The interviews used in the study were modified to provide a more structured interview onto which the algorithms to produce AGECAT diagnoses were mapped. AGECAT categorised individuals on an ordinal scale for O0 to O5 where O0 indicates no organic symptoms, O1–O2 indicate subclinical symptoms, and O3–O5 indicate case-level organic diagnosis. These were applied to the assessed sample at both the prevalence and incidence phases. All the records available from individuals unable to complete the full interview (*n* = 23, eight with dementia) including a structured vignette from interviewers, informant information, and interviewers' standardised observations were reviewed clinically and a clinical judgement made. Most of these cases were individuals with severe prevalent dementia, living in institutions for whom such limited information was available that there was insufficient data to run the algorithms (none of these [*n* = 18] actually survived to be in this analysis and are not included above). Any individuals who recovered from dementia (indicating an inaccurate diagnosis at one time point) were additionally checked for their true diagnosis status using interim interviews and the same information used above, as were all individuals suspected of having dementia, but with high MMSE scores (*n* = 7).

### Statistical Methods

Version 6.2 of the data was used for this analysis. Analysis has been undertaken using Stata version 7.0 [37].

#### Centre analysis

The study power calculations were based on overall centre estimates, not centre, age, and sex, because sample sizes would need to be much greater for such comparison. A single age-standardised incidence rate by centre is provided referenced to the 1991 age population of England and Wales [22].

#### Incidence rate calculation

Due to the complex nature of the design, individuals were included in the assessment phases on the basis of stratified random sampling that used the following factors: interview version, age group, and ability group (as detailed previously). Calculation of the incidence rate in two-phase studies is complicated by individuals in the incidence assessment interview (A2) having unknown prevalence diagnosis, unless a sensitivity of 100% at sampling is assumed. This assumption is not plausible in MRC CFAS because the cognitive threshold for 100% selection was quite low. The analysis presented here does not assume that the screening instruments are 100% sensitive. Some of the “new” cases found at the incidence assessment would have been prevalent cases of dementia had they received a prevalence assessment, and therefore, are not classified as incident cases.

A weighted pseudo-likelihood approach to the parameters of interest gives two maximum likelihood estimators that can be combined to calculate the incidence. The first estimator calculates the probability of not having dementia at the second wave, θ̂ . This probability is calculated using all individuals in the second wave assessments (both in A2 and C2). The second estimator calculates the proportion of cases of dementia at the second wave that already had dementia at the first wave, λ̂ (C2 only). This estimate is weighted to the original study population seen at the prevalence wave in order to represent those with dementia, but not assessed during this wave. These estimates can be combined to give a probability of being an incident case ϕ̂ = (1 − θ̂)(1 − λ̂) and then used to estimate the proportion of individuals with incident dementia in the absence of dementia at the first wave 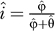 . An additional advantage of this method is that it enables updates of the prevalence estimates using the new two-year information [38].

Waves were planned to be 2 y apart, with time from prevalence screen to prevalence assessment being one month. The incidence rate is calculated as the proportion of individuals who have incident dementia, , divided by the mean time between waves (2 y) and presented as a rate per 1,000 person years. The 95% confidence interval of the incidence rates have been calculated by using 5,000 bias-corrected and accelerated bootstrap samples [39].

### Sensitivity Analysis

There are many different ways of approaching data in incidence studies. Two sensitivity analyses are presented here to investigate robustness.

#### Complete diagnosis analysis

A sensitivity analysis was carried out using only individuals who had two assessment interviews, 2 y apart. Individuals originally not having dementia at wave 1, but having it at wave 2 are “true” incident cases. This allows examination of bias in estimating the proportion of individuals with dementia newly assessed at wave 2 who were not assessed at prevalence. Using solely the individuals with complete case information (the A0 and C2 group), incidence estimates can be calculated directly and then back-weighted to the population at prevalence screen (using the same method as in the original prevalence paper [21]). This estimate is less precise than the method described already because it is based on fewer individuals, but provides information on bias and potential problems with the modified incidence technique presented above.

#### Inclusion of mortality

Individuals with dementia are known to be at greater risk of death than those without dementia of the same age and sex [40]. Individuals lost to follow-up due to death could therefore have been incident dementia cases, and not included in our analysis. To examine the impact this might have, a sensitivity analysis has been conducted by combining the known rates of dementia with an estimate in the literature of the excess of dementia seen in deceased individuals. A relative risk of dementia in deceased individuals of six in the 65–74 y age group, four in the 75–84 y age group and two in the 85 y and above has been used [41]. The analysis has not been adjusted for dropout due to refusal, as initial analysis of the dropout mechanisms shows no effect of dementia on refusal rates, after adjusting for age, sex, and cognitive ability [42]. The analysis here is already adjusted for age and sex, and the weighting adjusts for cognitive ability.

Hospital and general practice records were not systematically reviewed across sites because pilot studies in MRC CFAS Cambridgeshire showed that these are too inaccurate to act as proxies of actual state. Recording of known dementia on death certificates has been reported to be inaccurate in other countries [43] and provisional analysis in MRC CFAS appears to confirm this (M. Dewey, personal communication).

## Results

### The Sample

The response rates at baseline ranged from 71% to 79%, with 19% refusal rate. Figure 1 and Table 1 provide the audit trail of the sample from prevalence screen to incidence assessment (fuller details for each site are available on the study Web site, http://www-cfas.medschl.cam.ac.uk). Attrition through death and refusal can be seen to be the most important reasons for loss to follow-up, with 32% lost to follow-up; and key characteristics of those who drop out were that they were more likely to be male, older, cognitively impaired, functionally impaired, and living with others, with less education and fairly poor self-perceived health (see attrition paper for more details [42]). The sampling characteristics of the study are shown in Table 2; most of the incident cases arise from AGECAT 03+ and low or missing MMSE groups, and these groups had very large sampling fractions. The number of cases and a back-weighted estimate of the total number of cases are also shown.

**Table 1:**
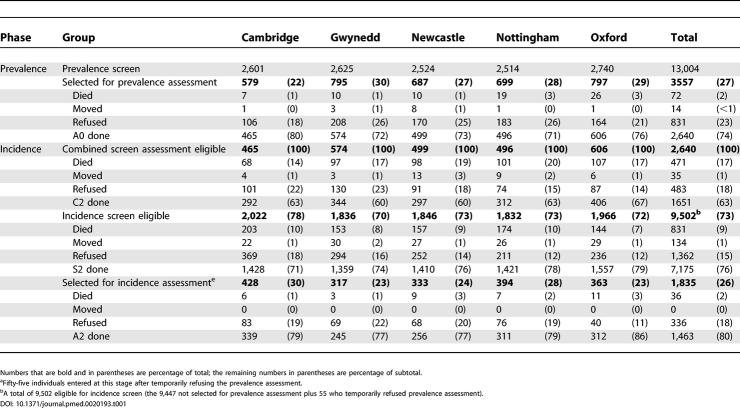
Numbers Included in the First Two Waves of MRC CFAS, Version 6.2

**Table 2:**
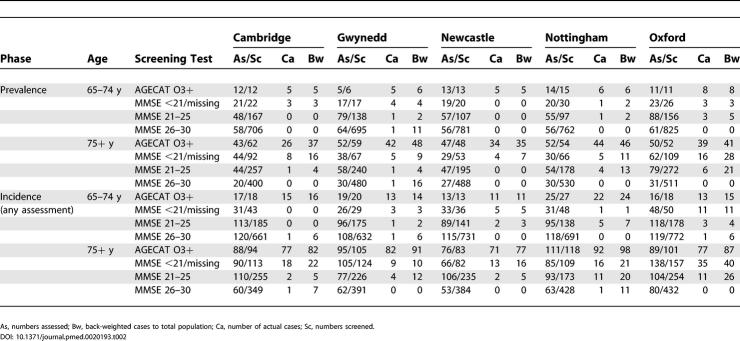
Numbers Assessed, Screened, Number of Actual Cases, and Back-Weighted Cases to Total Population by Sampling Strategy and Centre–Only Individuals Seen at Both Prevalence and Incidence Phase

### Incidence Data

In the complete study there were 20,165 person years of follow-up of individuals at risk in the study, 8,233 in men, and 11,932 in women. In the individuals that survived and returned for a follow-up interview at wave 2, there were 17,310 person years of follow-up of individuals at risk in the study, 7,115 in men, and 10,195 in women. There were fewer in the very old, with 355 person years at risk in the 90 y and over age group. A total of 8,826 individuals were re-seen at the wave 2 interviews who were initially in the first wave interview. Seven individuals not having dementia at prevalence were excluded because their dementia status at wave 2 was unknown, leaving 8,819 in the incidence analysis. There were 136 individuals at C2 who had not previously received a case-level diagnosis. In addition there were 212 cases identified at A2 whose prevalent dementia status was unknown. These figures when back-weighted to the total sample equate to 774 cases at wave 2, of whom about 404 initially had dementia, giving an estimated 370 incident cases. In checking case vignettes for possible misclassification of individuals with MMSE above standard cut points who were diagnosed with incident dementia, we found 35 individuals (out of 1,888) with MMSE at wave 2 above 21 (and 2 out of 895 with MMSE above 26) who had dementia at wave 2.

### Centre Analysis

Incidence rates by centre are shown in Table 3 and Figure 2. The rates in each of the centres do differ as with prevalence, but not in a systematic manner (meta-analysis test for heterogeneity *p* = 0.22).

**Figure 2:**
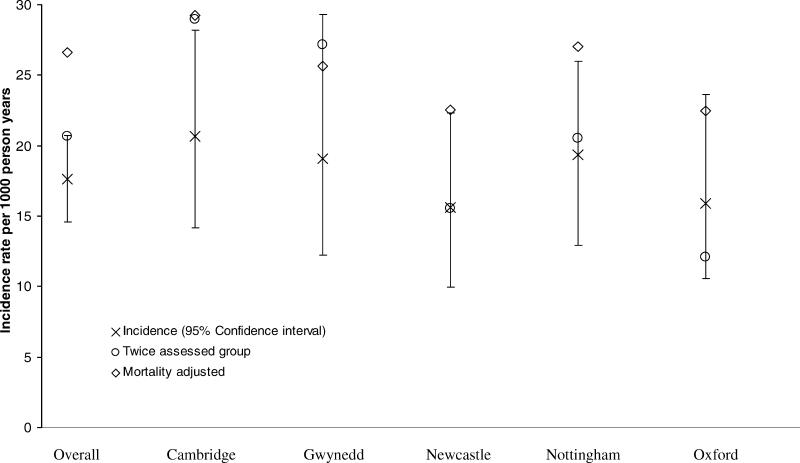
Centre Effects for the Three Different Incidence Models

**Table 3:**
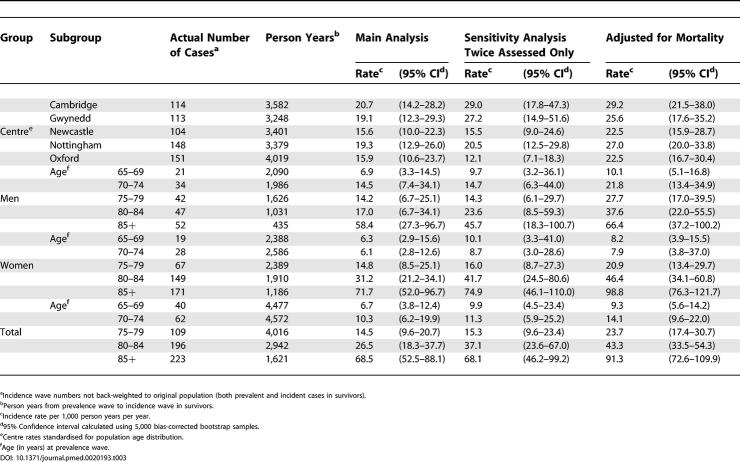
Number of Cases, Person Years, and Incidence Rate per 1,000 Person Years with 95% Confidence Interval

### Combined Incidence Analysis

Figure 3 shows the incidence rates per 1,000 person years by age at prevalence screen and by sex, and Table 3 gives more detail to the overall sex and age effects. Incidence increases consistently with age for both men and women, and the rates overall are similar for the two sexes. The population burden of these rates equates to approximately 180,000 new occurring dementia cases each year in England and Wales (95% confidence interval [CI], 105,000 to 325,000).

**Figure 3:**
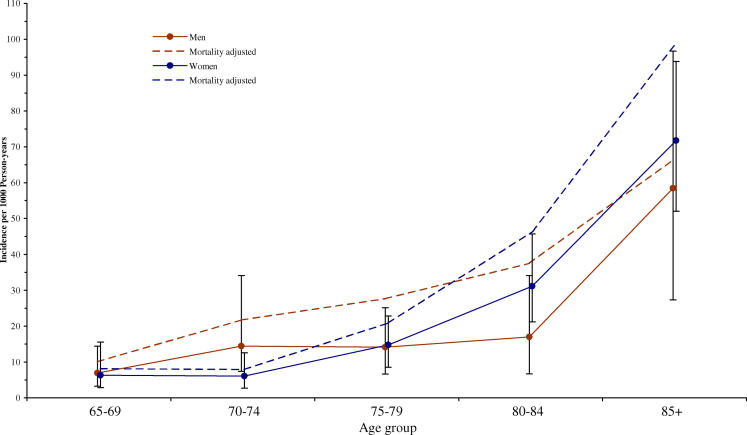
Incidence Rates (and 95% Confidence Intervals) by Age and Sex

### Sensitivity Analysis (Twice-Assessed Group)

Incidence rates calculated using the known incident cases and back weighting to the prevalence screen population show very similar rates. The confidence intervals around these estimates are wide (Table 3).

### Sensitivity Analysis (Mortality Effect)

As expected, rates adjusted for a mortality effect are higher than those without an adjustment, but only slightly. The potential bias from the deceased individuals has the most effect in men and on the estimate at age 75–79 y in which both the death and dementia rate are rising. Of the 1,219 individuals that died between wave 1 and wave 2, 220 already had dementia. Of the 750 with unknown dementia status prior to death, the majority were in groups where the chances of having prevalent dementia were clearly defined (107 [14%] with very high risk and 443 [59%] with very low risk). These figures indicate that the choice of 2 y between waves dramatically reduced the bias of dropout owing to death on the incidence estimate.

## Discussion

### Summary of Findings

The MRC CFA Study provides the first multi-site comparison of incidence rates in ethnically homogeneous populations within a country and within Europe using identical methodology. These are diverse sites with varying exposures of potential importance in dementia [14]. As in the prevalence study, no major differences were found across the sites. Incidence does reflect prevalence despite the variation in life expectancy across different regions in England and Wales. Thus these estimates provide robust estimates for the incidence of dementia in England and Wales for age and sex for the first time. They also provide robust estimates using methods that can be repeated across time for the investigation of cohort effects.

Gender effects were not found to be as marked as in many other studies, with very similar rates between men and women overall.

Incidence rates in both sexes rise very dramatically with age, also reported in our formal risk analysis which showed an odds ratio for the oldest versus youngest age groups of 23.6 (90+ vs. 65–69 y, 95% CI, 10.5–53.0). There is no evidence of the rates slowing in the oldest age groups, with sufficient person years at risk to provide robust estimates of rates up to the age of 90 y.

### Methodological Considerations

#### The sample

The samples followed here were geographically based, with reasonable response rates, similar or higher than most other comparable studies, and known attrition; and the base population was representative of the populations from which they were sampled including institutionalised respondents. This is in contrast to many dementia studies that do not provide robust population estimates because they either exclude institutionalised subjects or cannot weight with confidence for the local population.

#### Sample attrition

The greatest threat to the validity of the findings is the dropout experienced over the 2 y. This analysis does not take interim interviews into account, or any further sources of information, such as death certificates. However these are unlikely to influence the estimates in a substantial way, although they could reduce the variance seen. The results do not adjust for attrition between waves due to refusal, inability to contact, or moving from study area, which possibly makes them conservative, due to cognitively impaired individuals having higher drop-out rates [42].

#### Interviewer bias across sites

Consistent bias in interviewer ratings and responses across the sites could influence the centre findings, but this was controlled for through the national coordinator providing systematic training, regular quality control and highly structured interviews.

#### Diagnosis

The diagnosis used here was based on the AGECAT computerised algorithm [21]. The organicity section of AGECAT was found to be sensitive to depression in the screening interview, when used in isolation from the rest of the instrument. In the assessment interview, it was more stable and has shown good validity in a number of studies conducted in a variety of settings [44,45]. Its main advantage over standard clinical diagnosis is that interviews are conducted by interviewers trained to achieve reliable ratings on standardised items, which contribute to the computerised algorithm, reducing the likelihood of systematic bias thus providing consistency across sites and over time. Smaller studies can afford clinical assessment and have often used consensus diagnoses. These are extremely time consuming, and no cost-effectiveness studies exist to compare a formal standardised approach such as used here to provide evidence for efficiency of methods put against some form of clinical input. The one study published on GMS and AGECAT methods suggests that the computerised algorithmic approach is no more variable when compared with clinical consensus style assessments [46].

A small number of cases were diagnosed through clinical review of the records, largely from the prevalence phase; as explained in Methods, this was due to poverty of data rather than clinical uncertainty. There is no guarantee of consistency of consensus diagnosis over time, and thus the estimates provided here, although perhaps less clinically attractive, are robust enough for future comparison because the methodology is repeatable.

This analysis does not address clinical subtyping, but the series of donations in the neuropathological component from this study has provided the proportions of individuals having dementia with different particular pathologies. This analysis did reveal that Alzheimer-type pathology does predominate in the age groups studied here, but also that vascular disease is common and that both co-occur in most individuals [47].

#### Sensitivity analysis

Many biases can be introduced in such studies. Dropout and mortality are major reasons for concern. In the analysis presented here, the possible effects of mortality have been taken into account in the sensitivity analysis and do not provide substantially different results.

#### Statistical methods

The methods used here were developed specifically for this study; they therefore take into account the exact details of the study design. The methods can be generalised to any study of a chronic disease with a two-phase selection process. They are based however on weighting methods defined within strata; hence the use of many groups (e.g., age, sex, and centre) would have too much instability.

### Implications of Findings

Many studies with similar two-phase, two wave designs assume that those individuals who screen negative at baseline could not have dementia (e.g., Rotterdam [1] and Paquid, France [48,49]). In the MRC CFAS design, no such assumption was made, with sampling for assessment interviews from above the cut point. The analysis has taken into account, at both prevalence and incidence waves, that a small proportion of those above standard cut points (i.e., those who screen negative) actually had dementia. Therefore this analysis has fully adjusted for this type of error. It is reassuring that the results obtained by this method are of the same order of magnitude as in previously published studies, being broadly comparable with the combined re-analyses conducted by Jorm and Jolley [50]. The European combined analysis, which does not include these data, but those from the sister site, Liverpool, provides estimates that are also broadly similar; however MRC CFAS gives the highest results of all the studies. Figure 4 shows the comparison between the results obtained from the Jorm and Jolley meta-analysis [50] and the EURODEM group [20]. There is no marked heterogeneity between the United Kingdom and other European sites. MRC CFAS rates appear to be similar to those for the European definition of mild dementia. The EURODEM exercise provides almost identical estimates, apart from young age groups. The slightly higher estimates do suggest that studies that do not take all design features into account may slightly underestimate their rates. Small differences, particularly in the older ages, could give rise to large effects at a population level and have major implications for policy makers, although the differences at young ages could require changes in policy and care provision.

**Figure 4:**
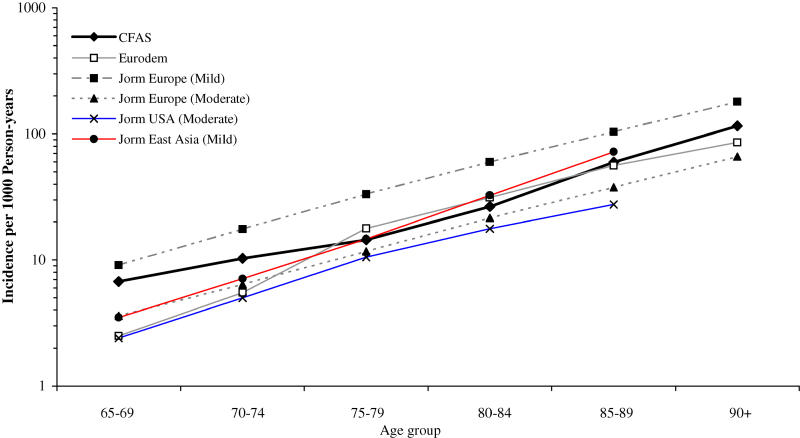
Incidence Rates in MRC CFAS Compared with Meta-Analysis Results

Lack of a decline in incidence in extreme age suggests that at the population level, there is not an extreme survivor effect as has been suggested previously based on prevalence rates [51]. This may be an artefact of the response rates, survival effects, and nature of previous populations sampled in these very old age groups. Although the combined analyses of incidence studies suggest that there is a gender effect, this is not found here. The male increase in life expectancy is likely therefore to be accompanied by substantial increases in the numbers of incident dementia in men as the average life expectancy moves towards the age at which the rates increase dramatically [52].

The methods used within this paper can be applied to any study of dementia, whether longitudinal or two-phase, as long as some form of follow-up has been attempted on everyone. The results do not assume that the individuals who screen negative do not have dementia, and are not sensitive to weighting assumptions in this analysis.

Although many risk factors have been described for dementia and Alzheimer's disease, there is no evidence within England and Wales that variation at the population level in these risk factors influences incidence in the population. There is no evidence of tailing off in the incidence rates for dementia in the oldest age groups, contrasting with previous suggestions in the literature that rates might be lower in the oldest age groups [51]. These findings have considerable implications for future planning nationally and internationally in the context of global ageing.

**Patient Summary**

**Background**

Dementia remains a condition without a cure. Dementia is defined as a progressive deterioration in cognitive abilities that interfere with ability to function independently. It is more common among older people, and a big health problem in aging societies. When describing how common dementia is, scientists use two measurements: the prevalence, meaning how many people have dementia at a particular time point or within a particular time period; and the incidence, meaning the number of new cases in a particular time period.

**Why Was This Study Done?**

Even though dementia is a big public health issue, we do not have good comparative data on its incidence or prevalence. This is mostly because different studies have used different definitions for dementia and different methods to collect data, and are thus not suitable for accurate comparisons. The British Medical Research Council funded this study to get data on dementia in the United Kingdom that were suitable to compare dementia incidence between men and women, different age groups, different locations, etc.

**What Did the Researchers Do and Find?**

The researchers studied dementia incidence in individuals from five different sites in England and Wales, two in rural areas and three in cities. In all sites, they studied over 1,000 people each from two age groups: between 65 and 74 y, and 75 y and over. They found that incidence rises with age among both men and women, and that this rise continues in the oldest age groups. There were no differences between the five sites.

**What Does This Mean?**

It seems that known differences in risk factors between some of the sites did not have a great effect. Likewise, this careful analysis did not reveal differences between men and women that had been suggested by other studies. Another previous suggestion, namely that incidence might fall again in the oldest age groups, was also not confirmed by the data here. The results will be important for future health care planning within the United Kingdom and in other countries with rapidly aging populations.

**Where Can I Find More Information Online?**

The following Web sites have relevant information.

The home page of the Medical Research Council Cognitive Function and Ageing Study:

http://www-cfas.medschl.cam.ac.uk/

MedlinePlus on dementia:

http://www.nlm.nih.gov/medlineplus/dementia.html

The American Geriatrics Society's Foundation for Health in Aging (search for dementia):

http://www.healthinaging.org/about/

The Alzheimer's Society (United Kingdom):

http://www.alzheimers.org.uk/

Alzheimer's Disease International (an international federation of national Alzheimer Associations):

http://www.alz.co.uk

Alzheimer's Association (United States):

http://www.alz.org/

## Abbreviations

A0: prevalence assessment interview
A2: incidence assessment interview
C2: combined screen and assessment interview
CI: confidence interval
GMS: Geriatric Mental State
MMSE: Mini-Mental State Examination
MRC CFAS: Medical Research Council Cognitive Function and Ageing Study
S0: prevalence screen interview
S2: incidence screen interview
