# Correction: The Incidence of Dementia in England and Wales: Findings from the Five Identical Sites of the MRC CFA Study

**DOI:** 10.1371/journal.pmed.0020389

**Published:** 2005-10-25

**Authors:** Fiona Matthews, Carol Brayne

In *PLoS Medicine*, volume 2, issue 8, DOI: 10.1371/journal.pmed.0020193

Some of the data presented in the Abstract and Results were incorrect.

The third sentence of the Methods and Findings section in the Abstract should read as follows: “Incidence rates rise with age, particularly above the age of 75 y, from 6.7 (95% confidence interval, 3.8–12.4) per 1,000 person years at age 65–69 y to 68.5 (95% confidence interval, 52.5–88.1) per 1,000 person years at age 85 y and above.” The fifth sentence should read as follows: “Hence, it is estimated that approximately 163,000 new cases of dementia occur in England and Wales each year.”

In Results, under “Combined Incidence Analysis,” the third sentence should read as follows: “The population burden of these rates equates to approximately 163,000 new occurring dementia cases each year in England and Wales (95% confidence interval [CI] 96,000 to 272,000).”

